# Risk factors for Graves' Orbitopathy in surgical patients—Results of a 10‐year retrospective study with review of the literature

**DOI:** 10.1002/edm2.210

**Published:** 2020-12-04

**Authors:** Navid Tabriz, Arved Gruben, Verena Uslar, Dirk Weyhe

**Affiliations:** ^1^ School of Medicine and Health Sciences University Hospital for Visceral Surgery Pius‐Hospital Oldenburg Carl von Ossietzky University Oldenburg Oldenburg Germany

**Keywords:** Graves' disease, multivariable analysis, thyroid surgery, TRAb

## Abstract

**Introduction:**

We investigated known (eg age, smoking, thyrotropin receptor autoantibody (TRAb)) and new risk factors (eg thyroid peroxidase autoantibodies (TPO‐Ab), thyroid size, or BMI) for Graves' disease (GD) and Graves' orbitopathy (GO), especially in combination with each other, to determine which factors play the most important role in the development of GO.

**Methods:**

From 2008 to 2018, *n* = 500 patients with GD were included in this retrospective single‐centre case‐control study. *N* = 231 (46%) had a GO and *n* = 269 (54%) showed no GO. Differences in risk factors were determined by Mann‐Whitney U and chi‐square test. Combined influences of factors were examined by multivariable logistic regression.

**Results:**

Age at first diagnosis of GD (OR = 1.043, *p* < .006), smoking status (OR = 2.64, *p* < .026) and TRAb (OR = 1.046, *p* < .01) had a significant impact on GO. The factors gender, TPO‐Ab titre, BMI, TSH titre, T3 and T4 were not significant.

**Conclusion:**

As it has been shown in univariate analyses, smoking, age and TRAb levels have a negative impact on the onset and course of GD and GO. Via multivariable regression, we could additionally show that smoking is the most important factor out of those analysed. TRAb might be a helpful surrogate parameter in the assessment of the progress of GO and therefore might be one factor in the decision‐making process for potential early operative surgery. With regard to the hitherto unclear role of BMI, thyroid size and TPO‐Ab in the course of GO, this study could not find any clinically relevant influence.

## Graphical abstract

We investigated known (eg, age, smoking, thyrotropin receptor autoantibody (TRAb)) and new risk factors (eg, thyroid peroxidase autoantibodies (TPO‐Ab), thyroid size, or BMI) for Graves' disease (GD) and Graves' orbitopathy (GO), especially in combination with each other, to determine which factors play the most important role in the development of GO in 500 patients with GD. Via multivariable regression, we could show, that smoking is the most important factor out of those analyzed. TRAb can be used as a surrogate parameter in the assessment of the progress of GO, thus helping in the decision‐making process for potential early operative surgery.

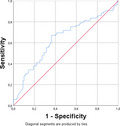

## 1 INTRODUCTION

Graves' orbitopathy (GO) is a potential concomitant autoimmune disorder in patients with Graves' disease (GD) which primarily targets the orbits by autoimmune processes leading to increased local formation of adipocytes.^1^ Its incidence is age‐related between 1–27/10.000/year with a peak between ages of 40 and 60 years. The prevalence is 10/10.000/year in Europe.^2^

GO can consequently result in several optical sensations like diplopy, dry eye syndrome or exophthalmos and can affect the optical nerve leading to blindness in rare cases.^3^ Thus, affected patients frequently report a decreased quality of life in addition to eye syndromes, and they often need psychological support.^4^ Therefore, the optimal therapy not only includes the treatment of GO but also the prevention or progression of symptoms by avoidance and control of risk factors.^5^ Well‐studied risk factors for GO include smoking, thyrotropin receptor autoantibody (TRAb) levels, age and sex^6^, ^7^, ^8^ But also risk factors such as radioiodine therapy (RJT) and hyper/hypothyroidism seem to aggravate the outcome in GO.^9^

To further investigate the influence of potential risk factors on GO, we conducted a retrospective single‐centre analysis of patients with GD with and without GO treated by surgery. As primary end‐point, the effect of known factors (eg age, gender, smoking habits, or TRAb) and potential new risk factors such as thyroid peroxidase antibody (TPO‐Ab) and body weight on probability of developing GO in GD was examined through a multifactorial regression model. The estimated effects were compared with the literature. Secondary end‐point was the calculation of a receiver operating characteristic (ROC) curve to determine the risk of GO development as a function of the TRAb titre.

## 2 MATERIALS AND METHODS

### 2.1 Study design

**FIGURE 1:**
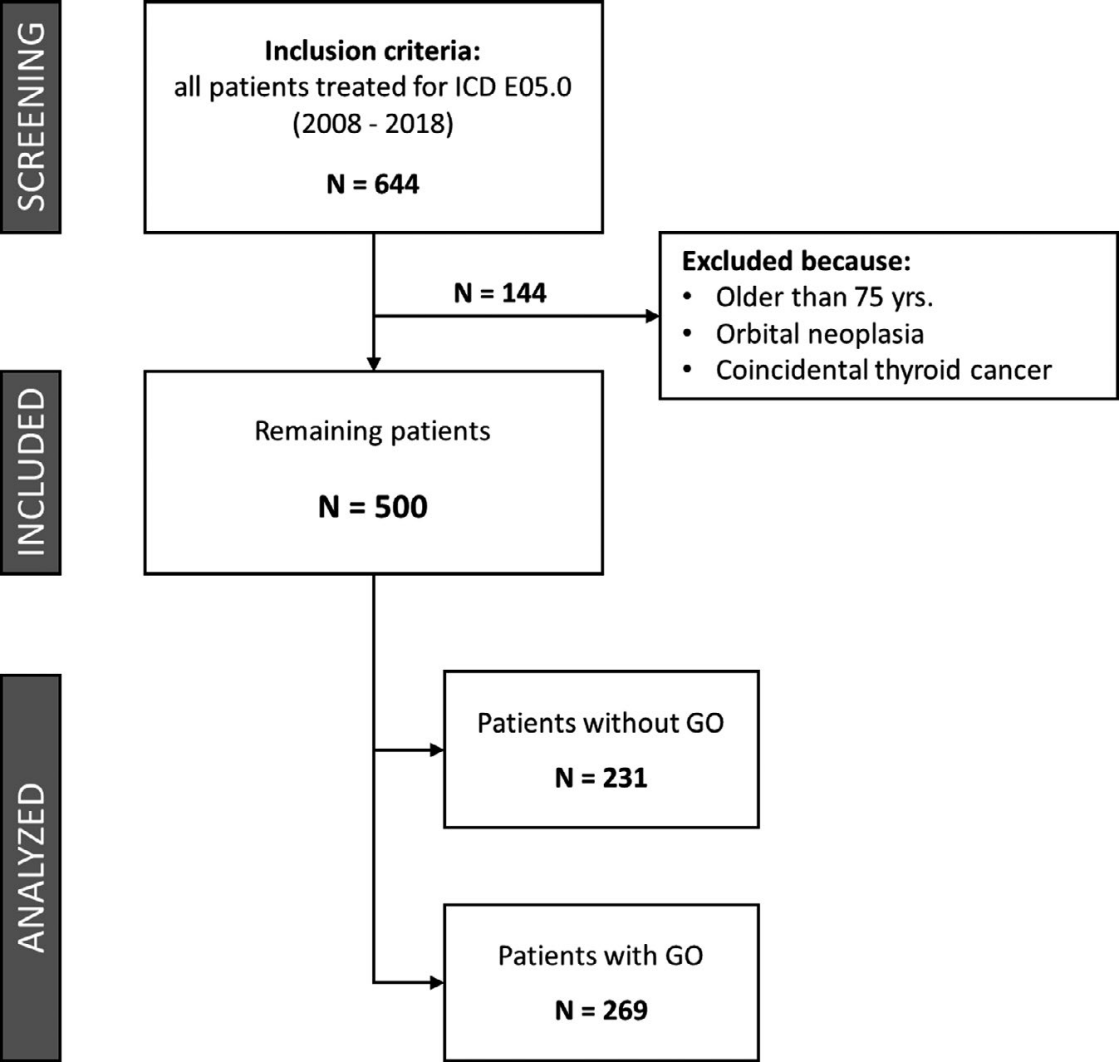
Study flow chart

The study was conducted as a single‐centre retrospective case‐control study and was approved by the Medical Ethics Committee of University of Oldenburg (reference number 2019‐012). Between 2008 and 2018, all patients with GD treated by total thyroidectomy due to persistent or relapse of hyperthyroidism at the University Clinic for Visceral Surgery, Pius Hospital Oldenburg, School of Medicine and Health Sciences, Carl von Ossietzky University Oldenburg, were retrospectively included. In patients where both RAI and surgery were possible options, surgery was performed at the patient's request. Initially, all patients with ICD E.05.0 were selected (*n* = 644; Figure 1). Patients older than 75 years, as well as patients with orbital neoplasia and/or coincidental thyroid cancer, were excluded. Eventually, a number of *n* = 500 patients could be included for further analysis. This collective was examined for the diagnosis of GD.

All cases with GO (group 1) were compared with the cases without GO (group 2) for following risk factors: age, sex, smoking status, body mass index (BMI), thyroid weight in the pathological findings, TRAb, thyroid‐stimulating hormone (TSH), TPO‐Ab. TSH, free triiodothyronine (fT3) and free tetraiodothyronine (fT4) were determined preoperatively while TRAb and TPO‐Ab were determined at time of initial diagnosis of GD when no thyrostatic medication was taken. Patients suffering from GO were assigned to the NOSPECS classification based on available ophthalmological records.

### 2.2 Statistical analysis

For data collection, Microsoft Excel, and for statistical assessment, SPSS 25 were used. For numerical data, averages and standard deviations, or medians and interquartile ranges (IQR) were calculated, depending on the distribution. Categorical data were presented as absolute and relative frequencies. Furthermore, for the two groups (patients with GO/patients without GO) differences in assessed factors were identified by the chi‐squared test (nominal scaled factors) or the Mann‐Whitney U test (ordinal scaled factors). The analysis for the influence of the combined factors was carried out by means of multiple logistic regression analysis with the binary dependent variable GO. The significance level was *p* < .05 at a 95% confidence interval, and false discovery rate was calculated to account for multiple testing where appropriate.^10^ In addition, odds ratios for the possible risk factors were determined and reported with the associated confidence intervals. A ROC curve was calculated to determine a cut‐off value for the TRAb titre. Specificity and sensitivity were included, and the area under the curve was determined. The ROC curve was created using SPSS 25, the study flow chart was created using Microsoft PowerPoint.

## 3 RESULTS

Of the *n* = 500 patients analysed, *n* = 231 [(46%); *n* = 189 (46%) female; *n* = 42 (47%) male; Table 1] had a GO, and in *n* = 269 patients [(54%); *n* = 221 (54%) female; *n* = 48 (54%) male] no GO was documented (n.s.). The mean age at time of operation was for women 39.2 ± *SD* 13.3 years and for men 41.1 ± *SD* 13.7 years (n.s.). For *n* = 434 patients, the age at time of primary diagnosis of GD could be recorded. Patients with GO were significantly older at initial diagnosis (Mann‐Whitney *U* test *U* = 19 986.5; *p* < .022). The grade of GO measured by NOSPECS Score was 2 ± ICQ 2 for female and 3 ± ICQ 2,5 for male (n.s.). The BMI, TSH and the size of resected tissue did not significantly differ between the groups.

**TABLE 1:** Patients' characteristics dependent on GO existence

	No Orbitopathy		Orbitopathy		*p*‐value^a^
	*n*	Median (IQR)	*n*	Median (IQR)	
Age diagnosis GD (years)	231	38 (26.3–47)	203	41 (30–51)	**.022**
Body mass index (kg^2^/m^2^)	180	25.1 (22.3–28.3)	152	24.8 (22.3–29.4)	.3667
Pathol.thyroid weight (g)	199	31 (25–46)	151	32 (22–48)	.70
TRAb (IU/L)	98	7.7 (3.5–15.2)	85	13 (6.1–33.3)	**.022**
TPO‐Ab (U/ml)	95	497 (75.6–2169)	76	360.7 (91.9–1601)	.638
TSH (mU/L)	266	0.01 (0.005–0.88)	228	0.02 (0.005–1.2)	.24
T3 (µg/L)	257	3.9 (3.0–5.6)	223	3.6 (2.7–5.1)	.198
T4 (ng/ml)	257	1.27 (0.9–1.7)	223	1.15 (2.1–2.3)	.3025

Abbreviations: IQR, Interquartile Range; TPO‐Ab, Thyroid Peroxidase Antibody; TRAb, Thyrotropin receptor autoantibody; TSH, Thyreotropin.

^a^ Mann‐Whitney *U* test; corrected after Benjamini and Hochberg.^10^

The bold values are significant results.

**FIGURE 2:**
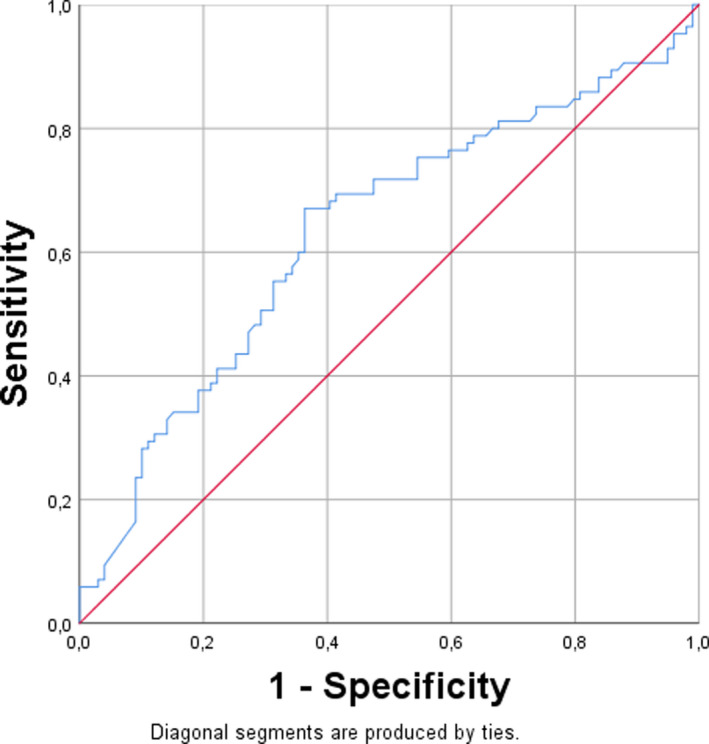
Result of the ROC analysis

Out of the measured serological values, only the TRAb showed a significant difference between the groups and TRAb was almost twice as high in patients with GO 13 IU/L vs. 7.7 IU/L (Mann‐Whitney *U* test *U* = 19 986.5; *p* < .022). For the TRAb titre, a cut‐off value was determined by ROC analysis. A balanced cut‐off value was reached at TRAb of 10.645 IU/L (Figure 2). Patients with TRAb titres higher than this cut‐off value had a greater risk for developing GO. The sensitivity was 0.65 and the specificity was 0.63. The area under the curve was 0.633.

The proportion of smokers was 42% overall, of which 63% had a GO. Compared to the group of nonsmokers of which only 34% had a GO, there was a significant difference (chi‐square (1) = 27.558, *p* < .001; Table 2).

**TABLE 2:** Frequency distribution for different risk factors depending on the occurrence of GO

	No Orbitopathy *n* = 269 (54%)	Orbitopathy *n* = 231 (46%)	Total	*p*‐value^a^
Sex				
Female	221 (54%)	189 (46%)	410 (82%)	.922
Male	48 (53%)	42 (47%)	90 (18%)	
Overweight				
BMI^1^ < 25 kg/m^2^	89 (56%)	69 (44%)	158 (47%)	.908
BMI^1^ > 25 kg/m^2^	93 (52%)	85 (48%)	178 (53%)	
BMI^1^ < 30	149 (55%)	121 (45%)	270 (80%)	.908
BMI^1^ > 30	33 (50%)	33 (50%)	66 (20%)	
Smoking status				
Nonsmoker	135 (66%)	70 (34%)	205 (58%)	**<.001**
Smoker	55 (37%)	92 (63%)	147 (42%)	
TPO‐Ab				
TPO‐Ab negative	21 (57%)	16 (43%)	37 (20%)	.966
TPO‐Ab positive	84 (57%)	63 (43%)	147 (80%)	

Abbreviations: BMI, Body Mass Index; TPO‐Ab, thyroid peroxidase antibody.

^a^ Chi^2^ Test; corrected after Benjamini and Hochberg.^10^

The bold values are significant results.

A multiple logistic regression was performed with the dependent variable orbitopathy, which included the following independent variables: age at initial diagnosis GD, smoking status, TRAb, sex, BMI, thyroid weight, TPO‐Ab, TSH, T3 and T4. A blockwise variable inclusion was chosen to check for possible correlations. The best model was achieved after inclusion of all parameters up to T4 (Chi2 28.836, *p* = .001; Table 3). The model correctly predicted 64.7% of patients who developed orbitopathy and 82.9% of those who developed none. The factors age at first diagnosis (OR = 1.043, *p* < .006), smoking status (OR = 2.64, *p* < .026) and TRAb (OR = 1.046, *p* < .01) were significant. Since the factor age might have been confounded by smoking habits or the TRAb titre changing over age, we performed a subgroup analysis with two age groups according to the mean age (≤49 years vs. >49 years). In the younger group, the ratio between smokers and nonsmokers was 60.4%–39.6%, in the older group 55.7%–44.3%. Mean value and standard deviation for the TRAb titre in the younger group were 23.0 ± 38.8, and 22.7 ± 54.4 in the older group.

**TABLE 3:** Result of the binary multiple logistic regression

	Regression coefficient	Standard error	Wald	*df*	*p*‐value	Odds Ratio	95% CI (Min & Max)	
Smoking	0.97	0.44	4.98	1	**.03**	2.64	1.13	6.19
Age diagnosis GD	0.04	0.02	7.44	1	**.01**	1.04	1.01	1.08
TRAb	0.04	0.02	6.67	1	**.01**	1.05	1.01	1.08
Sex	−0.74	0.64	1.31	1	.25	0.48	0.14	1.69
TPO‐Ab	0.00	0.00	1.10	1	.30	1.00	1.00	1.00
BMI	0.00	0.04	0.00	1	.99	1.00	0.93	1.08
TSH	0.10	0.14	0.52	1	.47	1.11	0.84	1.47
Thyroid weight	0.00	0.01	0.27	1	.60	1.00	0.99	1.02
T3	−0.06	0.08	0.64	1	.42	0.94	0.81	1.09
Constant	−2.20	1.46	2.26	1	.13	0.11		

Abbreviations: BMI, Body Mass Index; TPO‐Ab, thyroid peroxidase antibody; TRAb, Thyrotropin receptor autoantibody.

The bold values are significant results.

## 4 DISCUSSION

Nearly 20%–25% of patients with GD suffer from GO.^3^ This issue does not only concern optical symptoms but also impairs patients' quality of life. Therefore, an early and appropriate therapy of GD and orbitopathy according to the 2016 European guidelines is crucial.^5^ In our cohort, 46% of the patients with GD were affected by a GO. This amount is surely higher than the mentioned prevalence and is most likely due to the retrospective unicentric character of the study as patients affected by GO are introduced more often to the surgeon. It can also be assumed that the fact that we only included patients with surgical therapy created a certain bias. These patients have larger thyroid volumes, which in itself is a known risk factor for GO. In the context of this study, however, this collective was particularly interesting for us because it can provide information about which risk factors can still lead to the development of GO even after surgery.

Several risk factors for development and impairment of GO have been described, of which smoking seems to have the worst prognosis. In general, smoking patients with GD tend to have more (severe) GO, and smokers are more likely to have progression or de novo occurrence of GO after radioiodine treatment.^11^, ^12^ This fact can be emphasized by our results, as smokers are affected more than twice as often as nonsmokers by GO (odds ratio 2.64, 95% CI: 1.13–6.19) even if we cannot quantify the smoking behaviour, so that a correlation between the intensity of smoking and the extent of the GO could not be investigated. Smoking cessation is crucial in the therapy of GD irrespective of the presence/absence of GO.

Female patients are more likely to be affected by GD and GO in a 2–3:1 ratio.^13^, ^14^ In fact, in our study, the female‐to‐male ratio concerning the prevalence of GD was 4:1 and even higher than in the mentioned studies. A reason for the higher probability for women to receive surgical treatment could be the fact that a (planned) pregnancy is a contraindication for radioiodine therapy, and therefore, more women are assigned to surgery. Although autoimmune disorders like the GD are more common in women and GO seems to be more frequent in men, gender does not seem to have a significant impact on the development of GO in our study, as the ratio between patients with and without GO was approximately comparable for women and men. Confounding factors like smoking habits, even in different gender groups, or differences in ancestry should be considered in the interpretation of this results.^9^ Men with GD are usually affected by a more severe manifestation of GO and at a higher age than women.^14^, ^15^ We could not find any significant gender‐specific differences in the severity of GO regarding the NOSPECS Score. Since the NOSPECS Score could be calculated retrospectively only for 37 male patients, a larger number of cases would certainly be necessary to prove a gender‐specific effect.

Most of the patients develop a GO in the age range of 40–50.^16^ Patients with GO are significantly older at first diagnosis of GD (38 vs. 41 years), and these patients are at higher risk for developing GO (OR 1.04, 95% CI 1.01–1.08). Although this effect was not particularly clear, our data are in line with Khong et al, who also found that older age at first diagnosis of GD increases the risk of GO (OR 1.12).^17^ In two further studies, similar results were confirmed.^8^, ^18^ The subgroup analysis performed to control for possible confounding shows that the age effect is not due to an unequal distribution of smokers or patients with different TRAb titres in the age groups formed.

TRAb plays a crucial role in the pathogenesis of GD and GO, and it is assumed that its level correlates with clinical outcome like relapse of GD and severity of GO.^6^, ^19^ The level of TRAb could therefore be used as a helpful adjuvant in the decision‐making process of early definitive therapy like RAI or surgery as first choice in cases of TRAb > 12 IU/L at diagnosis.^20^ In patients with GO, it has been shown that higher TRAb titres correlate with more severe GO in the entire course of the disease. Therefore, patients with TRAb titres above a certain cut‐off value, indicating a severe course of GO, could benefit from a modified or prolonged thyrostatic or immunosuppressive therapy and shorter control intervals.^6^ In our study, a correlation of TRAb with the presence of GO could be confirmed. Patients with TRAb titres higher than 10.65 IU/L had twice as high a risk of developing a GO (OR 1.05, 95% KI 1.01–1.08. However, it has to be noted that the sensitivity 0.65, specificity 0.63 and AUC 0.63 are not high. Certainly, the level of TRAb helps in the clinical and therapeutic management of patients with GD and GO. Thus, future studies are needed to evaluate if the TRAb titre indeed can be used as a surrogate parameter in assessing the risk of relapse of hyperthyrosis in GD or development/aggravation of GO. Recently, it has been shown, that irrespective of the presence of GO, GD patients could benefit from a thyroidectomy compared to RAI with regard to cardiac arrythmia.^21^ Therefore, an (early) thyroidectomy may be discussed in patients with GO, TRAb > 10 IU/L, or cardiac symptoms.

In contrast to TRAb which is specific for GD, TPO‐Ab can be detected in GD as a facultative parameter.^22^ TPO can be expressed in the orbit of patients with GO^23^, ^24^ and a paediatric study has shown an association between high TPO‐Ab levels and ocular involvement.^25^ In contrast, in adults, a correlation between low TPO‐Ab titre and GO was found.^26^ We could not find any difference in TPO‐Ab levels between patients with and without GO. The role of the TPO‐Ab in GO remains unclear due to the low number of studies and needs further investigation.

In addition to the TRAb titre, thyroid size seems to play a role in predicting relapse in GD as well as the severity of GO.^27^, ^28^ As we cannot present any differences in the weight of resected thyroid tissue between the groups, this fact cannot be emphasized by our findings. But it should be considered that the design and inclusion criteria of our study might contain some bias as only patients for thyroidectomy were included who might have larger thyroid sizes than patients for RAI. This might explain the fact, that we could not establish thyroid size as a risk factor in our study.

Total and low‐density lipoprotein (LDL)‐cholesterol seems to correlate with the presence and activity of GO, suggesting a role of cholesterol in the development of GO.^29^ In how far higher BMI can influence the GO has not been investigated. We could not find any effect of BMI on GO even if subgroup analysis with BMI > 25 m^2^/kg or BMI > 30 m^2^/kg are performed. Adiposity does not seem to influence GO in general. However, a high BMI does not necessarily have to be associated with a high LDL level, so that a conclusive statement about the influence of LDL based on the surrogate parameter BMI cannot be made, and a differentiation of cholesterol subtypes could not be done in our retrospective cohort.

## 5 CONCLUSIONS

Often, risk analyses only take one risk factor into account. By looking at several risk factors in combination, we could confirm that smoking, TRAb levels, and age at diagnosis can be used as surrogate parameters to calculate the risk of the development of GO, at least in patients scheduled for surgery. The role of TPO‐Ab, BMI and thyroid size remains unclear. As smoking habit has the greatest influence on GO, patients should be strongly advised to quit smoking. TRAb titres can be used as prognosis and follow‐up parameter in GD and GO. Patients with TRAb levels > 10 IU/L seem to have an increased risk for GO and should therefore be closely monitored for a potential early definitive therapy.

## CONFLICT OF INTEREST

The authors declare that they have no conflict of interest.

## AUTHORS CONTRIBUTIONS

All authors contributed to the study conception and design. Material preparation and data collection were performed by Arved Gruben. Analysis was performed by Verena Uslar. The first draft of the manuscript was written by Navid Tabriz and all authors commented on previous versions of the manuscript. All authors read and approved the final manuscript.

## ETHICAL APPROVAL

The study was conducted in accordance with the ethical standards of the institutional committee (Medical Ethics Commission of the Carl von Ossietzky University Oldenburg, reference number: 2019‐012) and with the 1964 Helsinki declaration and its later amendments.

## Data Availability

The data sets generated during and/or analysed during the current study are available from the corresponding author on reasonable request.

## References

[1] Garrity JA , Bahn RS . Pathogenesis of graves ophthalmopathy: implications for prediction, prevention, and treatment. Am J Ophthalmol. 2006;142(1):147‐153.e2. 16815265 10.1016/j.ajo.2006.02.047 PMC3960010

[2] Perros P , Hegedüs L , Bartalena L , et al. Graves' orbitopathy as a rare disease in Europe: a European Group on Graves' Orbitopathy (EUGOGO) position statement. Orphanet J Rare Dis. 2017;12(1):1‐6. 28427469 10.1186/s13023-017-0625-1 PMC5397790

[3] Bartalena L , Fatourechi V . Extrathyroidal manifestations of Graves' disease: a 2014 update. J Endocrinol Invest. 2014;37(8):691‐700. 24913238 10.1007/s40618-014-0097-2

[4] Kahaly G , Petrak F , Hardt J , Pitz S , Egle U . Psychosocial morbidity of Graves' orbitopathy. Clin Endocrinol. 2005;63(4):395‐402. 10.1111/j.1365-2265.2005.02352.x 16181231

[5] Bartalena L , Baldeschi L , Boboridis K , et al. The 2016 European Thyroid Association/European Group on Graves' orbitopathy guidelines for the management of GRAVES' orbitopathy. Eur Thyroid J. 2016;5(1):9‐26. 27099835 10.1159/000443828 PMC4836120

[6] Eckstein AK , Plicht M , Lax H , et al. Thyrotropin receptor autoantibodies are independent risk factors for Graves' ophthalmopathy and help to predict severity and outcome of the disease. J Clin Endocrinol Metab. 2006;91(9):3464‐3470. 16835285 10.1210/jc.2005-2813

[7] Thornton J , Kelly S , Harrison R , Edwards R . Cigarette smoking and thyroid eye disease: a systematic review. Eye. 2007;21(9):1135‐1145. 16980921 10.1038/sj.eye.6702603

[8] Perros P , Crombie A , Matthews J , Kendall‐Taylor P . Age and gender influence the severity of thyroid‐associated ophthalmopathy: a study of 101 patients attending a combined thyroid‐eye clinic. Clin Endocrinol. 1993;38(4):367‐372. 10.1111/j.1365-2265.1993.tb00516.x 8319368

[9] Stan MN , Bahn RS . Risk factors for development or deterioration of Graves' ophthalmopathy. Thyroid. 2010;20(7):777‐783. 20578901 10.1089/thy.2010.1634 PMC3357079

[10] Benjamini Y , Hochberg Y . Controlling the false discovery rate: a practical and powerful approach to multiple testing. J Roy Stat Soc: Ser B (Methodol). 1995;57(1):289‐300.

[11] Bartalena L , Marcocci C , Bogazzi F , et al. Relation between therapy for hyperthyroidism and the course of Graves' ophthalmopathy. N Engl J Med. 1998;338(2):73‐78. 9420337 10.1056/NEJM199801083380201

[12] Prummel MF , Wiersinga WM . Smoking and risk of Graves' disease. JAMA. 1993;269(4):479‐482. 8419666

[13] Burch HB , Wartofsky L . Graves' ophthalmopathy: current concepts regarding pathogenesis and management. Endocr Rev. 1993;14(6):747‐793. 8119236 10.1210/edrv-14-6-747

[14] Kendler DL , Lippa J , Rootman J . The initial clinical characteristics of Graves' orbitopathy vary with age and sex. Arch Ophthalmol. 1993;111(2):197‐201. 8431156 10.1001/archopht.1993.01090020051022

[15] Lim SL , Lim AKE , Mumtaz M , Hussein E , Wan Bebakar WM , Khir AS . Prevalence, risk factors, and clinical features of thyroid‐associated ophthalmopathy in multiethnic Malaysian patients with Graves' disease. Thyroid. 2008;18(12):1297‐1301. 19012471 10.1089/thy.2008.0044

[16] Wiersinga WM , Bartalena L . Epidemiology and prevention of Graves' ophthalmopathy. Thyroid. 2002;12(10):855‐860. 12487767 10.1089/105072502761016476

[17] Khong JJ , Finch S , De Silva C , et al. Risk factors for Graves' orbitopathy; the Australian thyroid‐associated orbitopathy research (ATOR) study. J Clin Endocrinol Metab. 2016;101(7):2711‐2720. 27055083 10.1210/jc.2015-4294

[18] Laurberg P , Berman DC , Bülow Pedersen I , Andersen S , Carlé A . Incidence and clinical presentation of moderate to severe Graves' orbitopathy in a Danish population before and after iodine fortification of salt. J Clin Endocrinol Metab. 2012;97(7):2325‐2332. 22518849 10.1210/jc.2012-1275 PMC3387399

[19] Ponto KA , Kanitz M , Olivo PD , Pitz S , Pfeiffer N , Kahaly GJ . Clinical relevance of thyroid‐stimulating immunoglobulins in Graves' ophthalmopathy. Ophthalmology. 2011;118(11):2279‐2285. 21684605 10.1016/j.ophtha.2011.03.030

[20] Hesarghatta Shyamasunder A , Abraham P . Measuring TSH receptor antibody to influence treatment choices in Graves' disease. Clin Endocrinol. 2017;86(5):652‐657. 10.1111/cen.13327 28295509

[21] Gibson A , Dave A , Johnson C , Kotwal A , Fingeret AL . Cardiovascular outcomes of thyroidectomy or radioactive iodine ablation for Graves' disease. J Surg Res. 2020;256:486‐491. 32798996 10.1016/j.jss.2020.07.020

[22] Ross DS , Burch HB , Cooper DS , et al. 2016 American Thyroid Association guidelines for diagnosis and management of hyperthyroidism and other causes of thyrotoxicosis. Thyroid. 2016;26(10):1343‐1421. 27521067 10.1089/thy.2016.0229

[23] Fernando R , Lu Y , Atkins SJ , Mester T , Branham K , Smith TJ . Expression of thyrotropin receptor, thyroglobulin, sodium‐iodide symporter, and thyroperoxidase by fibrocytes depends on AIRE. J Clin Endocrinol Metab. 2014;99(7):E1236‐E1244. 24708100 10.1210/jc.2013-4271 PMC4079309

[24] Lai OF , Zaiden N , Goh SS , et al. Detection of thyroid peroxidase mRNA and protein in orbital tissue. Eur J Endocrinol. 2006;155(2):213‐218. 16868133 10.1530/eje.1.02205

[25] Lee JH , Park SH , Koh DG , Suh BK . Thyroid peroxidase antibody positivity and triiodothyronine levels are associated with pediatric Graves' ophthalmopathy. World J Pediatr. 2014;10(2):155‐159. 24668239 10.1007/s12519-014-0476-y

[26] Lantz M , Planck T , Åsman P , Hallengren B . Increased TRAb and/or low anti‐TPO titers at diagnosis of Graves' disease are associated with an increased risk of developing ophthalmopathy after onset. Exp Clin Endocrinol Diabetes. 2014;122(02):113‐117. 24554511 10.1055/s-0033-1363193

[27] Profilo MA , Sisti E , Marcocci C , et al. Thyroid volume and severity of Graves' orbitopathy. Thyroid. 2013;23(1):97‐102. 23088654 10.1089/thy.2012.0379

[28] Shi H , Sheng R , Hu Y , et al. Risk factors for the relapse of Graves' disease treated with antithyroid drugs: a systematic review and meta‐analysis. Clinical. 2020;42(4):662‐675.e4. 10.1016/j.clinthera.2020.01.022 32139177

[29] Sabini E , Mazzi B , Profilo MA , et al. High serum cholesterol is a novel risk factor for Graves' orbitopathy: results of a cross‐sectional study. Thyroid. 2018;28(3):386‐394. 29336220 10.1089/thy.2017.0430

